# Single nucleotide polymorphism array‐based signature of low hypodiploidy in acute lymphoblastic leukemia

**DOI:** 10.1002/gcc.22956

**Published:** 2021-05-17

**Authors:** Thomas Creasey, Amir Enshaei, Karin Nebral, Claire Schwab, Kathryn Watts, Gavin Cuthbert, Ajay Vora, John Moppett, Christine J. Harrison, Adele K. Fielding, Oskar A. Haas, Anthony V. Moorman

**Affiliations:** ^1^ Leukaemia Research Cytogenetics Group, Translational and Clinical Research Institute Newcastle University Newcastle upon Tyne UK; ^2^ Department of Clinical Genetics Children's Cancer Research Institute Vienna Austria; ^3^ Northern Genetics Service The Newcastle‐upon‐Tyne Hospitals NHS Foundation Trust, Institute of Genetic Medicine, International Centre for Life Newcastle upon Tyne UK; ^4^ Haematology and Oncology Department Great Ormond Street Hospital London UK; ^5^ Paediatric Haematology Department Bristol Royal Hospital for Children Bristol UK; ^6^ Department of Haematology UCL Cancer Institute London UK

**Keywords:** acute lymphoblastic leukemia, cytogenetics, hypodiploid, SNP array

## Abstract

Low hypodiploidy (30–39 chromosomes) is one of the most prevalent genetic subtypes among adults with ALL and is associated with a very poor outcome. Low hypodiploid clones can often undergo a chromosomal doubling generating a near‐triploid clone (60–78 chromosomes). When cytogenetic techniques detect a near triploid clone, a diagnostic challenge may ensue in differentiating presumed duplicated low hypodiploidy from good risk high hyperdiploid ALL (51–67 chromosomes). We used single‐nucleotide polymorphism (SNP) arrays to analyze low hypodiploid/near triploid (HoTr) (*n* = 48) and high hyperdiploid (HeH) (*n* = 40) cases. In addition to standard analysis, we derived log2 ratios for entire chromosomes enabling us to analyze the cohort using machine‐learning techniques. Low hypodiploid and near triploid cases clustered together and separately from high hyperdiploid samples. Using these approaches, we also identified three cases with 50–60 chromosomes, originally called as HeH, which were, in fact, HoTr and two cases incorrectly called as HoTr. *TP53* mutation analysis supported the new classification of all cases tested. Next, we constructed a classification and regression tree model for predicting ploidy status with chromosomes 1, 7, and 14 being the key discriminators. The classifier correctly identified 47/50 (94%) HoTr cases. We validated the classifier using an independent cohort of 44 cases where it correctly called 7/7 (100%) low hypodiploid cases. The results of this study suggest that HoTr is more frequent among older adults with ALL than previously estimated and that SNP array analysis should accompany cytogenetics where possible. The classifier can assist where SNP array patterns are challenging to interpret.

## 1 INTRODUCTION

Acute lymphoblastic leukemia (ALL) is characterized by recurrent chromosomal abnormalities within the leukaemic blasts that are prognostic even in the era of measurable residual disease‐adapted treatment protocols.^1^, ^2^, ^3^, ^4^ Large non‐random ploidy shifts define three distinct primary genetic subtypes of ALL: High hyperdiploidy (51–67 chromosomes), near‐haploidy (23–29 chromosomes), and low hypodiploidy (30–39 chromosomes).^5^ High hyperdiploidy (HeH) occurs in one‐third of childhood cases and is associated with a favorable outcome.^4^ In contrast, near‐haploidy and low hypodiploidy are rare in childhood ALL (<2% each) and are associated with a very poor outcome.^6^, ^7^, ^8^ The frequency of low hypodiploidy increases with age, occurring in >5% adult cases and is the second most prevalent chromosomal abnormality (>10%) among older adults (>60 years); whereas near‐haploidy is virtually non‐existent in adult ALL.^2^, ^9^, ^10^, ^11^ In adults, low hypodiploidy is associated with a very poor outcome even when the patients are treated as high risk.^2^, ^3^, ^10^, ^12^

The pattern of chromosomal loss in low hypodiploidy is variable but non‐random. Chromosomes 3, 7, 15, 16, 17 are lost most frequently while chromosome 21 is always retained.^5^ Cases of low hypodiploidy commonly present with a co‐existing near‐triploid clone with 60–78 chromosomes,^9^, ^12^ and the genetic subgroup is therefore termed HoTr hereafter. The pattern of chromosomal loss/gain and the duplication of structurally rearranged chromosomes provide evidence the two clones are related and that low hypodiploidy is the primary event.^13^ The mechanism by which the low hypodiploid clone doubles is thought to be a process of chromosomal endo‐reduplication without subsequent cytokinesis thereby creating leukaemic blasts with a near triploid karyotype of 60–78 chromosomes. Cytogenetic analysis of 115 paediatric HoTr cases from the Children's Oncology Group revealed the duplicated clone to be present in 76 (66%) cases.^7^ In some cases, cytogenetic analysis reveals only a near‐triploid clone with a pattern of chromosome gain (i.e., frequent tetrasomies and duplicated structural abnormalities) suggestive of a low hypodiploid origin.^7^, ^12^ In such cases, distinguishing between HoTr and HeH rests on the modal chromosome number and pattern of chromosome gains; potentially generating a diagnostic dilemma.^5^, ^7^, ^9^ A very high proportion (90%) of HoTr cases harbor pathogenic *TP53* mutations which are usually germline in paediatric cases.^14^, ^15^, ^16^, ^17^ Although HoTr and near‐haploidy share some features (e.g., chromosome loss and clonal doubling) the distinct mutational profile and age distribution indicate that they are distinct subgroups.^5^, ^9^, ^14^, ^18^

The rapid and accurate identification of HoTr is crucial in both adult and childhood ALL to assign patients to the optimal therapy. Historically, cytogenetic and FISH analyses have formed the basis of leukemia genetic testing but recently genomic techniques have emerged and are used to supplement or replace traditional methods.^19^, ^20^ SNP arrays are very useful for detecting large ploidy shifts and loss of heterozygosity (LOH).^19^, ^20^, ^21^ LOH is a common finding in neoplastic clones and can be a manifestation of monosomy or multiple copies of the same chromosome.^22^, ^23^ The hallmark of HoTr by SNP array is widespread LOH in all chromosomes at the lower copy number state,^13^, ^24^ reflecting LOH arising from chromosomal loss. A similar pattern is seen in cases presenting with a near triploid clone alone, consistent with the prevailing hypothesis that this has arisen by endoreduplication.^12^ In comparison, HeH ALL typically shows preserved heterozygosity in the majority of chromosomes with single additional maternal or paternal homologues in most chromosomes at the higher copy number state.^25^ LOH can be seen in HeH but affected chromosomes have at least the same copy number state as preserved heterodisomies as chromosomal loss has not occurred.^25^ Despite the wealth of SNP array data that exists for ALL, few cases of HoTr have been included due to the bias toward paediatric ALL and the rarity of the subgroup.^19^, ^20^ This study combines cytogenetic and SNP array data to highlight the challenge of detecting this clinically relevant subgroup. We report a novel approach to analyzing SNP array patterns from highly aneuploid samples and in addition, develop and validate a classifier to help distinguish between HoTr and HeH using SNP array patterns when accompanying cytogenetic analysis is not available.

## 2 METHODS

We identified patients and samples from the Leukaemia Research Cytogenetics Group (LRCG) database, as previously described,^26^ and from the Northern Genetics Service, Newcastle‐upon‐Tyne Hospitals NHS Foundation Trust. Patients were enrolled on UKALL14, UKALL60+, UKALLXII, UKALL2011, or UKALL2003 trials giving informed written consent for treatment and genetic studies. Cytogenetic and FISH analyses were performed in and reported from regional genetic laboratories across the UK. Karyotypes and surplus material were collected for central review and additional testing. Karyotypes were described according to the International System for Human Cytogenetic Nomenclature (ISCN) and, for consistency and clarity, were always reported relative to the diploid (2*n*) state. Fixed cells or DNA from pre‐treatment diagnostic bone marrow were used for all analyses reported in this study; except where explicitly stated otherwise. SNP arrays were performed using the Illumina CytoSNP 850k (Illumina, San Diego, CA, USA) or Affymetrix Cytoscan HD array (Affymetrix, Santa Clara, CA, USA) in accordance with the manufacturers' protocols. Briefly, oligonucleotide probes were hybridized to regions across the genome generating log2 ratios of observed to expected probe intensity from internal platform‐specific reference datasets, as previously described.^22^, ^24^, ^27^, ^28^ Illumina‐generated IDAT files were first processed using the GenomeStudio 2.0 (Illumina, San Diego, CA, USA), then loaded into Nexus Copy Number 10 (Biodiscovery, El Segundo, CA, USA) (Supplementary Methods). Affymetrix CEL files were directly loaded to Nexus. Default automated array normalization and systematic correction processes were performed according to the manufacturer's protocol.

### 2.1 Creation of whole‐chromosome copy number segments

All SNP array analyses were performed using the Nexus. Microarray intensities were median centered with positive or negative deflections representing relative gains or losses of genetic material respectively. A standard analysis of SNP array patterns was performed in Nexus by examining log2 ratio and B‐allele frequency traces independently of cytogenetics.^21^ In isolation, SNP arrays cannot resolve exact copy number states, particularly in samples with mixed clonal populations as all cellular context is lost. Therefore, each SNP array was assigned a descriptive label of (a) widespread LOH in chromosomes at the lower copy number state (LOH‐LCN), (b) preserved heterozygosity with copy number gains (HET‐CNG) or (c) insufficiently clear to call without additional information (Inconclusive). Copy number segments spanning the length of individual chromosomes 1–22 were then created and the median log2 ratio of each whole chromosome segment was automatically computed by Nexus and extracted for subsequent analyses. Sex chromosomes were excluded to ensure consistency between male and female patients. The degree of positive/negative deflection in log2 ratios within a SNP array is influenced by tumor purity, intra‐tumoral heterogeneity, and SNP array platform. To account for this inter‐sample variability, individual sample standardization of the 22 whole chromosomal log2 ratios was performed to mean of 0 and SD of 1 in R (version 4.0.3)^29^ using the R‐package BBmisc (Supplementary Methods). These standardized whole chromosome log2 ratios were then used for all subsequent clustering and classification analyses.

### 2.2 Unsupervised clustering of standardized whole chromosome log2 ratios

To assess whether standardized whole chromosomal log2 ratios produced distinct low hypodiploid, near triploid and high hyperdiploid signatures, unsupervised hierarchical clustering, and principle components analysis (PCA) were performed using the R‐packages ComplexHeatmap^30^ and prcomp,^31^ respectively, (code available at https://github.com/tcreasey/ALL_ploidy_classifier.git). R‐package FSelector^29^ was used to identify the whole chromosomal log2 ratios that contributed the most information (information gain) to the separation of the clusters. SNP array findings were then used to resolve any discrepancies between the cytogenetic diagnosis and the clustering analyses, to establish the most plausible ploidy subgroup.

### 2.3 *TP53* sequencing

For additional confirmation where SNP array findings conflicted with cytogenetics, *TP53* was sequenced in selected samples. A SureSelect XT2 kit (Agilent, Santa Clara, CA, USA) was used to capture coding regions of genes frequently mutated in ALL (Supplementary Table 1). Sample DNA was amplified using a REPLI‐g mini kit (Qiagen, Hilden, Germany) and libraries were prepared according to the manufacturer's protocol and sequenced on the NextSeq 550 instrument (Illumina) using 100bp paired‐end chemistry. Results were analyzed using the GATK Best Practices Workflow and Ensembl VEP^32^ (Supplementary Methods).

### 2.4 Decision tree classifier

A diagnostic classifier was developed based on a decision tree model (Supplementary Methods). Together with the HoTr and HeH cases, SNP arrays from an unselected cohort of other (“non‐ploidy”) patients were included and whole chromosome log2 ratios were derived and standardized as described above. This cohort consisted of 72 samples broadly representative of adult ALL and comprised *BCR‐ABL1* (*n* = 33), B‐other (*n* = 31), *KMT2A*‐rearranged (*n* = 5), *TCF3‐PBX1* (*n* = 1), and T‐ALL (*n* = 2) cases. To ensure the most accurate genetic diagnosis was being entered into the model, each case was classified as HoTr, HeH, or non‐ploidy using all available information from SNP arrays, cytogenetics, and *TP53* results where relevant. Using R‐package rpart,^33^ a classification and regression tree (CART) analysis was performed and a decision tree delineated using standardized chromosomal log2 ratios as variables to predict the ploidy group. The model was internally validated using 10‐fold cross validation in r‐package caret^34^ (Supplementary Methods, code available at https://github.com/tcreasey/ALL_ploidy_classifier.git).

### 2.5 External validation of the classifier

The classifier was externally validated using SNP array data from a cohort of 29 childhood ALL samples from Children's Cancer Research Institute (Vienna, Austria). The cohort comprised near haploidy (*n* = 8), HoTr (*n* = 7), HeH (*n* = 7), *ETV6‐RUNX1* (*n* = 2), *TCF3‐PBX1* (*n* = 1), *KMT2‐AFF1* (*n* = 1), B‐other ALL (*n* = 3). SNP arrays were performed and analyzed using the Affymetrix Cytoscan HD array and Chromosome Analysis Suite (ChAS) (Affymetrix, Santa Clara, CA, USA). KN extracted whole chromosome log2 ratios for each chromosome and sent the data blind to TC. TC standardized the data as described above before using the classifier to call each case as HoTr, HeH, or non‐ploidy based on standardized chromosomal log2 ratios alone. Results were returned to KN who un‐blinded the data.

## 3 RESULTS

### 3.1 Patient demographics, cytogenetics and SNP array interpretation

**FIGURE 1:**
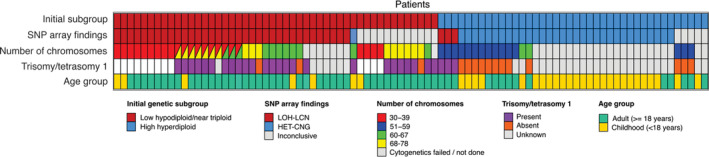
Patient demographics and cytogenetic characteristics. Patient samples were obtained from patients enrolled in UKALL14 (*n* = 40), UKALL2011 (*n* = 11), UKALL60+ (*n* = 6), UKALLXII (*n* = 6), and UKALL2003 (*n* = 3) clinical trials as well as local non‐trial cases (*n* = 22). Number of chromosomes has been divided into 30–39 (low hypodiploidy), 51–59 (high hyperdiploidy), 60–67 (high hyperdiploidy and near triploidy overlap), and 68–78 (near triploidy)

Our initial cohort comprised 88 cases identified as HoTr (*n* = 48) or HeH (*n* = 40) at initial diagnosis by either cytogenetics/FISH (*n* = 73) or SNP array (*n* = 15) by accredited diagnostic cytogenetic laboratories across the UK (Supplementary Table 2). Of those with karyotypes available (*n* = 57), additional structural chromosomal abnormalities were present in 39% (9/23) of low hypodiploid, 65% (11/17) of near triploid, and 41% (7/17) of high hyperdiploid clones. There were 55 adults and 33 children/adolescents. Although our cohort is selected in favor of HoTr cases, it is noteworthy that these patients were older within both the adult and paediatric cohorts: Mean 54.6 versus 44.6 years (p = 0.004) and 13.9 versus 4.7 years (p < 0.001); reflecting the disparate age‐specific frequencies of the subtypes (Figure 1).

**FIGURE 2:**
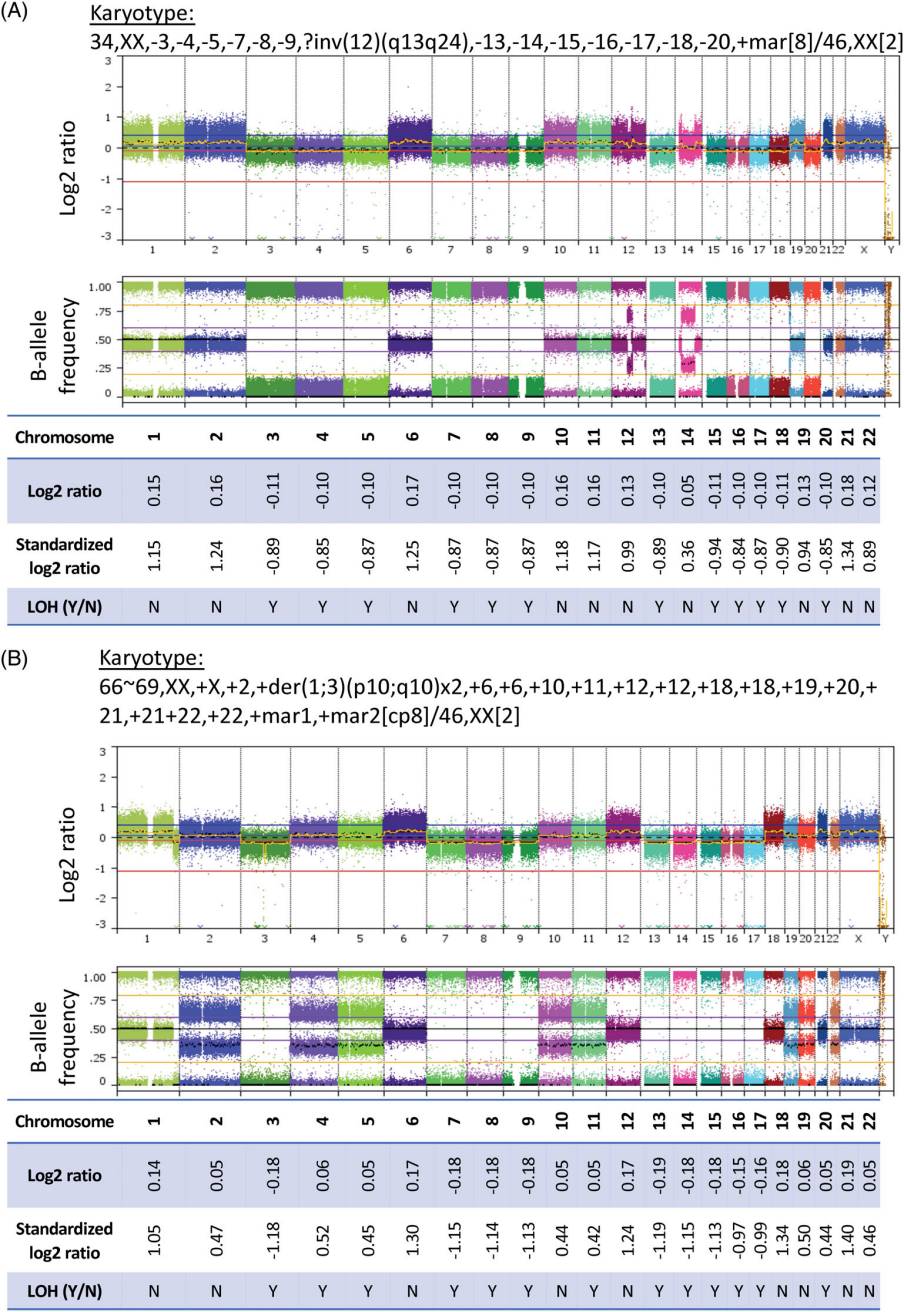
Single‐nucleotide polymorphism (SNP) arrays of low hypodiploid (A) and near triploid cases (B) (whole genome view of SNP arrays and whole chromosome log2 ratios, shown to 2 decimal places). (A) Example of a low hypodiploid case (#27069, blast percentage 96%) where only a low hypodiploid clone was detected on karyotype. Reduced log2 ratios are seen in chromosomes with complete loss of heterozygosity (LOH) on B‐allele frequency trace (LOH‐lower copy number [LCN]) and elevated log2 ratios in chromosomes with preserved disomic pattern of SNPs on B‐allele frequency. (B) Example case (#25437, blast percentage 88%) where only a near triploid clone was detected on karyotype. Reduced log2 ratios are seen in chromosomes with complete LOH on B‐allele frequency trace (LOH‐LCN) and elevated log2 ratios in chromosomes without LOH

Standard analysis of SNP arrays was performed on all 88 samples. In 32 cases, the pattern of HET‐CNG observed by SNP array and cytogenetics was consistent with the classic HeH profile (Supplementary Figures 1 and 2). The SNP array pattern of a further 35 cases exhibited LOH‐LCN, which was consistent with the HoTr profile (Figure 2 and Supplementary Figure 3). Among these 35 cases, cytogenetic analysis revealed a low hypodiploid, near‐triploid or both clones in 9, 9, and 10 cases, respectively. In two of the cases where cytogenetics only detected a near‐triploid clone, FISH identified a small low hypodiploid clone (#25614 and #27537, Supplementary Table 2). Cytogenetic analysis had either failed or was not done on the remaining seven cases (Figure 1). Interpretation of the SNP arrays for the remaining 21 cases led to a conclusion that contradicted cytogenetic analysis (*n* = 4) or was insufficiently clear to call without additional information (inconclusive) (*n* = 17). The inconclusive cases by SNP array had either a diagnostic karyotype (*n* = 14) or FISH analyses (*n* = 3) that had been used to assign the genetic subgroup (Supplementary Table 2).

**FIGURE 3:**
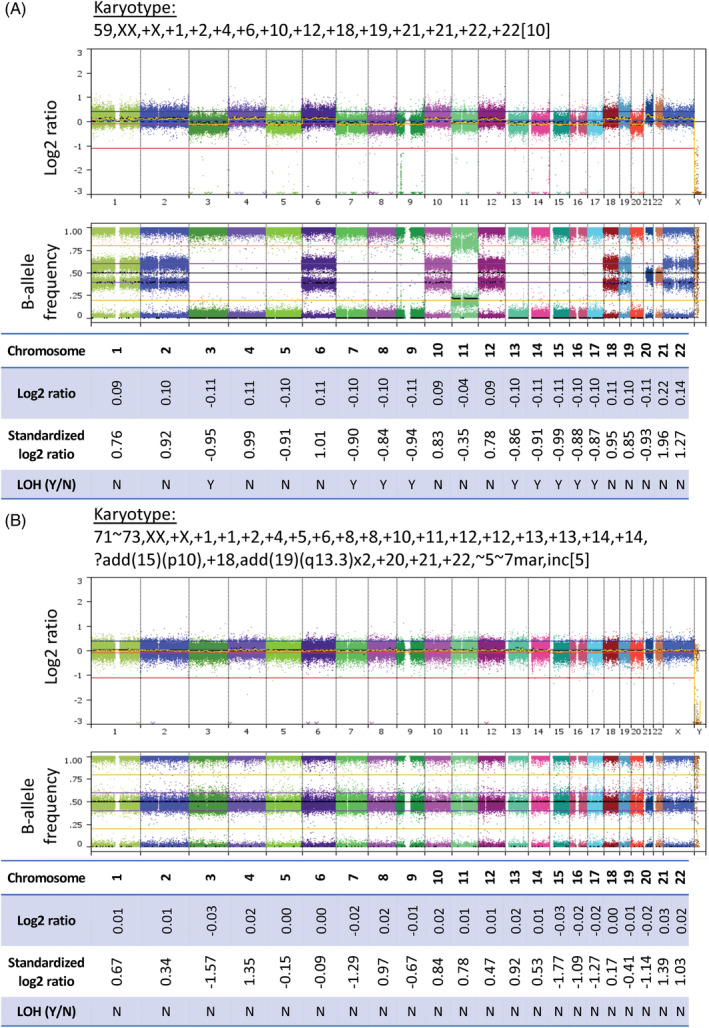
Single‐nucleotide polymorphism (SNP) arrays of cytogenetically misclassified (A) and visually inconclusive (B) cases (whole genome view of SNP arrays and whole chromosome log2 ratios, shown to 2 decimal places). (A) Example case (#27478, blast percentage 88%) cytogenetically classified as high hyperdiploidy with conflicting SNP array profile. SNP array demonstrates complete loss of heterozygosity (LOH) of chromosomes with the lowest copy number state (LOH‐lower copy number [LCN]). Other chromosomes show a trisomic complement of SNPs. Overall, the pattern observed is similar to that seen in hypodiploid/near triploid (HoTr) cases, contradicting initial cytogenetic subgroup despite modal chromosome number. (B) Example near triploid case (#28056, blast percentage 90%) with an inconclusive SNP array. The appearances would typically be associated with non‐leukaemic DNA contamination, although blast percentage in the diagnostic sample was high. The karyotype contains five tetrasomies and a duplicated structural abnormality consistent with HoTr. Although the log2 ratio and B‐allele frequency traces appear almost normal, the whole chromosome log2 ratio of chromosomes 3, 7, 15, 16, 17, 19, and 20 (which are frequently monosomic in low hypodiplody) is reduced. When standardized, whole chromosome log2 ratios correctly clustered with the HoTr cases (Figure 4)

Three cases (#26910, #27478, and #29491) initially classified as HeH by cytogenetics had a SNP array profile displaying widespread whole chromosomal LOH‐LCN (Table 1, Figure 3(A) and Supplementary Figures 4, 5). Interestingly all three had a modal chromosome number below the usual threshold considered for HoTr, namely 60 chromosomes.^5^, ^12^ Moreover, all three patients harbored pathogenic *TP53* mutations, a hallmark of HoTr (Table 1 and Supplementary Table 3). The *TP53* variants identified are reported in the Catalogue of Somatic Mutations in Cancer^35^ and were missense mutations affecting the DNA binding domain (*TP53* p.P151S and *TP53* p.R282W) and a nonsense mutation in the C‐domain (*TP53* p.K305*).

**TABLE 1:** Details of high hyperdiploid cases which clustered with low hypodiploid and near triploid cases or vice versa by unsupervised hierarchical clustering analysis of standardized whole chromosome log2 ratios

Patient ID	Age (years)	Abnormal karyotype	Subgroup by				Mutations	Outcome
			Cytogenetics	SNP array analysis	SNP array clustering	Decision tree node		
26 910	43	54~56,XY,+1,add (2)(q3)x2,+3,add (3)(q2),+5,+6,?del (6)(q?2),+10,+11,+14,+?16,+18,+2mar,inc[cp8]	High hyperdiploid	LOH‐LCN	HoTr	HoTr	*TP53* p.P151S	Died in CR1 within 1 year
27 478	58	59,XX,+X,+1,+2,+4,+6,+10,+12,+18,+19,+21,+21,+22,+22[10]	High hyperdiploid	LOH‐LCN	HoTr	HoTr	*TP53* p.R282W	Relapsed and died within 2 years
29 491	51	58~59,XY,+?X,+1,+2,+6,add (8)(q2)x2,+10,+11,+12,+12,+14,idic (15)(p1),+18,add (18)(p1),+19,+21,+21,+22,+mar,inc[cp10]	High hyperdiploid	LOH‐LCN	HoTr	HoTr	*TP53* p.K305*	CR1 (4 months)
24 805	46	53,XX,+5,+6,+10,+11,+20,+21,i (21)(q10),+22[8]	High hyperdiploid	Inconclusive	HoTr	Non‐ploidy	Not done	Died in CR1 within 1 year
27 058	7	64~66,XX,+X,+add (1)(p?2),+3,+4,+5,+6,+8,+10,+11,+12,+14,+14,+17,+18,+19,+20,+21,+21,+22,+mar[cp9]	Near triploid	HET‐CNG	High hyperdiploid	High hyperdiploid	Not done	CR1 (2 years)
28 893	27	75~80,XY,+X,+Y,+Y,+Y,+1,+1,+2,+2,+3,+4,+5,+5,+6,+7,+8,+9,+10,+11,+12,+13,+14,+14,+15,+15,+16,+16,+17,+17,+18,+18,+19,+19,+20,+20,+21,+21,+22,+22[cp4]	Near triploid	Inconclusive	High hyperdiploid	HoTr	*IGH‐CRLF2JAK2 p.T875NTP53* not mutated	CR1 (1 year)

*Note*: Three cases with cytogenetic classification of HeH showed widespread LOH‐LCN, consistent with HoTr. This was further confirmed by *TP53* mutations in all three cases. One case with a near triploid karyotype showed HET‐CNG on SNP array, consistent with HeH.

Abbreviations: HeH, high hyperdiploid; HET‐CNG, heterozygosity with copy number gains; HoTr, hypodiploid/near triploid; LCN, lower copy number; LOH, loss of heterozygosity.

One case (#27058) classified as HoTr by cytogenetics had a SNP array displaying the HET‐CNG pattern, more suggestive of HeH (Table 1, Supplementary Figure 6). The remaining 17 cases had inconclusive SNP array profiles (Figure 3(B)).

### 3.2 Clustering and classification of cases using SNP array data

**FIGURE 4:**
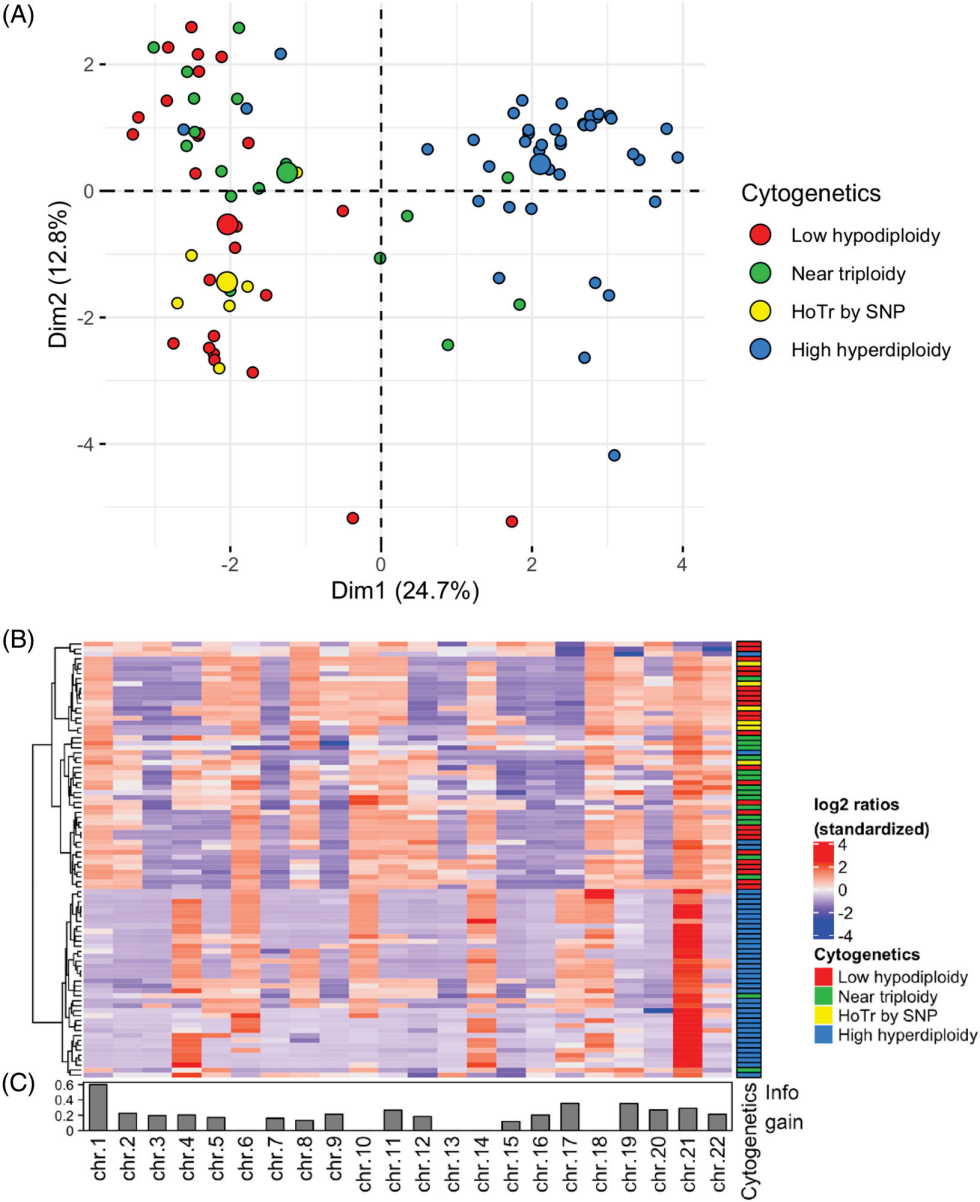
Unsupervised clustering of cases by standardized whole chromosome log2 ratios. Principal component analysis (A) and unsupervised hierarchical clustering as a heatmap (B) demonstrate clustering of low hypodiploid and near triploid cases separately from high hyperdiploid cases. Information contributed by each chromosome (information gain) displayed as a bar chart underneath (C). Cases within the incorrect cluster based on initial cytogenetic classification are detailed in Table 1

Unsupervised hierarchical clustering and principal component analysis (PCA) performed on the standardized whole chromosome log2 ratios (Supplementary Table 4) demonstrated that cases with low hypodiploid and/or near triploid clones clustered together (Figure 4), supporting the observation these are biologically related. Importantly, these HoTr cases clustered separately from HeH cases (Figure 4). Four cases classified by cytogenetics as HeH clustered with HoTr samples. Reassuringly three of these (#26910, #27478, and #29491) had been identified as having widespread LOH‐LCN and harbored pathogenic *TP53* mutations (Table 1); while the fourth case (#24805) had an inconclusive SNP result with a largely normal profile (Supplementary Figure 7). Among two cases (#27058, #28893) cytogenetically classified as HoTr that clustered with HeH cases, one (#27058) showed clear HET‐CNG on SNP array analysis (Supplementary Figure 6) and one (#28893) had an inconclusive SNP array profile (Supplementary Figure 8) but was found to harbor an *IGH‐CRLF2* gene rearrangement by FISH and a *JAK2* mutation, reflecting an alternative primary genetic abnormality.

### 3.3 Development and validation of ploidy classifier

**FIGURE 5:**
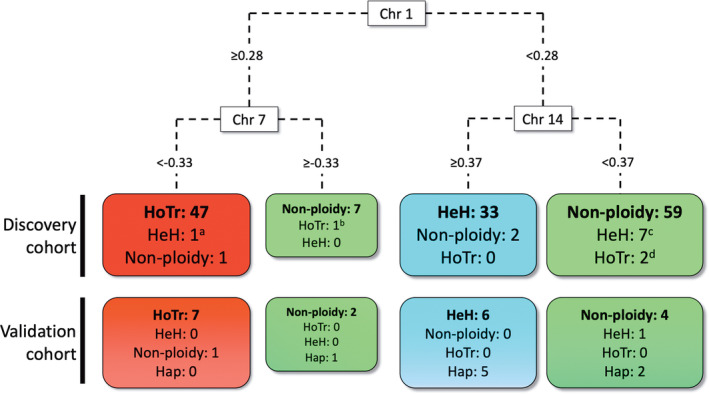
Decision tree for assigning cases to a genetic ploidy subgroup using standardized whole chromosome log2 ratios of chromosome 1, 7, and 14. Single‐nucleotide polymorphism (SNP) arrays with standardized log2 ratios for chromosome 1≥0.28 and chromosome 7 <− 0.33 had a 94% probability of being hypodiploid/near triploid (HoTr) cases. Cases with a standardized log2 ratios <0.28 for chromosome 1 and ≥0.37 for chromosome 14 had 94% probability of being high hyperdiploid (HeH). Cases where the log2 ratio was <0.28 for chromosome 1 and <0.27 for chromosome 14, had an 87% probability of the absence of major ploidy shift. Importantly, these three scenarios accounted for 95% of the patients in the dataset. A total of 11 cases called by cytogenetics and/or SNP array as having ploidy shifts were incorrectly assigned by the decision tree: (A) This patient had high hyperdiploidy and t (9;22)/*BCR‐ABL1*, which is recognized to have a different pattern of chromosomal gains from primary high hyperdiploidy;^36^ (B) Although this patient had a low hypodiploid karyotype with − 7, there was unbalanced translocation between the long arms of chromosome 6 and 7; (C) 4/7 cases failed cytogenetics while none of the remaining three cases had a + 14; (D) Karyotypes had been classed as HoTr by cytogenetics but SNP array analysis was inconclusive

To explore whether whole chromosome log2 ratios could be used to develop a ploidy classifier, we performed a CART analysis with an additional cohort of 72 patient samples spanning genetic subgroups lacking a primary ploidy shift. Prior to running the CART analysis, we re‐classified the four confirmed discrepant cases (#26910, #27478, #29491, and #27058) (Table 1) in line with SNP array and *TP53* findings. We also re‐classified the case with *IGH‐CRLF2* (#28893) into the non‐ploidy subgroup as the underlying primary genetic lesion was clearly distinct from both HoTr and HeH. Thus, the final CART analysis cohort comprised 50 HoTr, 41 HeH (including three with both *BCR‐ABL1* and HeH) and 69 non‐ploidy patients (Supplementary Table 2). A decision tree based on the complete dataset (*n* = 160) was derived from the CART analysis and identified the log2 ratios of chromosomes 1, 7, and 14 as the key discriminators of the three subgroups (Figure 5). Using these standardized log2 ratios, cases could be delineated into one of four terminal nodes: One each for HoTr and HeH and two for the non‐ploidy cases. The majority of HoTr cases (47/50, 94%) were correctly placed into the HoTr group, while three cases were placed into non‐ploidy groups. Similarly, the majority of HeH cases (33/41, 80%) were correctly assigned to the HeH node. Importantly, for diagnostic practice, chromosome 1 was a very powerful discriminator between HoTr and HeH ALL, and accurately segregated 97% (88/91) of cases with a ploidy shift. Our data show that if cytogenetic analysis or DNA index identify a hyperdiploid clone, the standardized log2 ratio of chromosome 1 (> or <0.28) can extremely reliably discriminate the biologically distinct HoTr and HeH entities (Figure 5). Importantly, our dataset included two HeH cases with dup (1q) (#28195 and #M18/968), which is a recognized structural abnormality in HeH ALL.^19^ Reassuringly, despite the resulting positive deflection in the standardized log2 ratio of chromosome 1, this remained <0.28, and these cases were therefore not misclassified as HoTr by the decision tree.

The model was then validated internally using 10‐fold cross validation and delivered an overall average accuracy of 0.79 (95% confidence interval 0.72–0.85) across all three ploidy classes with a precision of 0.91, 0.68, and 0.77 for HoTr, HeH, and non‐ploidy cases, respectively, and recall of 0.86, 0.78, and 0.74 for HoTr, HeH, and non‐ploidy cases, respectively.

The classifier was validated using an independent cohort of 29 samples analyzed using the Affymetrix Cytoscan HD platform. Individual whole chromosome log2 ratios were extracted, standardized and assessed using the classifier with the ploidy status blinded. The validation cohort included HoTr, HeH, and non‐ploidy cases, along with near haploid samples (Supplementary Table 5). The classifier correctly identified 7/7 cases with HoTr (Figure 5). The majority of HeH cases (6/7) and non‐ploidy cases (4/5) were also assigned to the correct group. Most of the near haploid cases (5/8) were classified into the HeH group which is logical given the discovery cohort did not include this entity and, in the majority, the duplicated near haploid clone included two copies of chromosome 1 and four copies of chromosome 14. This resulted in standardized log2 ratios <0.28 for chromosome 1 and >0.37 for chromosome 14; and an HeH call.

## 4 DISCUSSION

In this study, we present one of the largest SNP array cohorts to date of patients with HoTr ALL. Our observations show that HoTr may present with 50–60 chromosomes (as few as 54 chromosomes in our cohort), approaching the lower limit of the range for HeH. We have identified three cases with <60 chromosomes where the SNP array pattern was indicative of HoTr rather than HeH (Figure 3A and Supplementary Figures 4, 5). Crucially all three cases harbored a pathogenic *TP53* mutation which is the hallmark of this entity. We acknowledge that we did not show direct cytogenetic evidence of the presence of a low hypodiploid clone and that LOH is also well described in HeH,^25^ where it may occur as a result of chromosomal mis‐segregation during mitosis.^23^ Nonetheless, the LOH observed in these cases was extensive and affected the typical chromosomes lost in low hypodiploidy. Moreover, LOH was consistently seen in chromosomes with the lowest log2 ratios (LOH‐LCN), and those with preserved heterozygosity always had higher log2 ratios, suggesting these chromosomes initially became monosomic before duplicating. Interestingly, however, the modal chromosome number in these cases coupled with the high number of trisomies does question the prevailing hypothesis regarding the mechanism by which these karyotypes arise. Nonetheless the presence of a *TP53* mutation in these cases supports grouping and treating patients with such clones alongside patients with overt low hypodiploidy.

The samples and cases included in this study were selected on the availability of DNA and SNP array results but the age profile of the HoTr group does reflect the underlying epidemiology. Therefore, it is not possible to calculate the true proportion or incidence of misclassified cases from this study. However, we know that the frequency of HoTr increases with age, so these findings are particularly relevant in adult ALL and suggest the true frequency of this subgroup is higher than previously estimated. Indeed, we note all three cases initially misclassified as HeH, were adults >40 years old at diagnosis, suggesting HoTr may be even more common than currently appreciated in older patients. In addition, these findings may also explain the lack of consensus regarding the prognostic impact of HeH in adults.^36^

We used a novel approach to analyze SNP array patterns by deriving whole chromosome log2 ratios for each chromosome and, using the standardized data, performed cluster analysis and PCA across the HoTr and HeH cohort. The results confirmed that low hypodiploid and near triploid samples cluster together as expected, and separately from HeH samples, with chromosome 1 being the most discriminating factor for distinguishing between the two clusters (Figure 4).

SNP array analysis is performed on a fixed amount of DNA rather than a fixed number of cells, so exact multiples of cellular DNA content would all result in the same pattern on the microarray. This means that resolving the copy number state of individual chromosomes in samples that potentially contain a low hypodiploid clone and a separate inexactly duplicated clone is not possible when this cellular context is loss. As such, centering the log2 ratio to an assumed single diploid level is inappropriate as chromosomes are present in more than one copy number state in the sample. Although some studies have developed methods for normalization of aneuploid genomes, particularly with admixed non‐tumor DNA, these are based on the assumption of a single aneuploid tumor population at a constant ploidy level.^37^, ^38^ As shown, this is very frequently not the case in HoTr cases. However, within each sample, relative over or under‐representation of genomic loci can be deduced based on relative positive or negative deflections in the log2 ratio respectively. We applied this principle to entire chromosomes to derive whole chromosome log2 ratios, which we then standardized to a consistent scale for all samples. Importantly, we confirmed that no SNP array platform or batch effect was seen in the standardized whole chromosome log2 ratios (Supplementary Figure 9), supporting that our standardization permits comparison between samples assayed using different SNP array platforms and with varying tumor purity.

Using the standardized whole chromosome log2 ratios, we employed CART analysis to develop a classifier which we subsequently validated using an external blinded cohort. The classifier can accurately distinguish HoTr from HeH, and non‐ploidy samples (Figure 5). This contrasted with the outcome of the descriptive SNP array analysis where 17/88 cases had an inconclusive SNP array profile making the recognition of a specific ploidy pattern difficult. It is not clear why DNA from these 17 samples did not produce clear SNP array profiles but in three cases the DNA had been extracted from fixed cell suspension and in a further nine cases samples had been stored for >2 years prior to performing SNP arrays, potentially leading to noisy profiles. Alternatively, near normal SNP array patterns are often encountered when the leukaemic DNA content is low, although this did not seem to be the case with the majority of samples (Supplementary Table 2). However, importantly, these “real‐world” analysis issues did not hamper the reliability of our classifier, which was still able to accurately delineate cases lacking a clear diagnostic SNP array profile by standard visual analysis. Of these otherwise uninterpretable cases by standard SNP array analysis, our classifier was able to successfully resolve the ploidy status in 71% (12/17), including 10/12 HoTr cases (Supplementary Table 2). A limitation of our discovery cohort was the lack of near haploid samples whereas the validation cohort included eight such cases. These were included to identify whether they clustered with any other subgroups. The results confirm that the classifier is, as intended, specific for HoTr and further highlights that HoTr and near haploidy are distinct entities.^9^ Indeed, we have shown that, as expected, the classifier cannot be used to identify near haploidy. Although we recognize the importance of identifying this subtype in paediatric patients, we consider that the main strength of the classifier lies in accurately discerning HoTr cases from other genetic subgroups (precision 0.91, recall 0.86). In particular, we identified that chromosome 1 is consistently relatively over‐represented in HoTr compared with HeH ALL samples (Figure 5 and Supplementary Figures 10 and 11) and is the most discriminatory predictor to differentiate these two ploidy subgroups. In the absence of cytogenetics, log2 ratios of key chromosomes (1, 7, and 14) offer valuable information to resolve the genetic ploidy subgroup of a sample, even when visual interpretation of the SNP array is inconclusive. Current SNP array analysis software (e.g., Nexus or Affymetrix Chromosome Analysis Suite) can be used to derive whole chromosome log2 ratios, which can then be standardized as described, to support accurate genetic risk stratification in diagnostic genetic laboratories (Supplementary Figure 12). The classifier is relatively simple to use and, given the prognostic importance of HoTr, should be used whenever the results of a SNP array are ambiguous.

This study highlights the challenges in diagnosing this enigmatic genetic subtype. Ideally SNP array profiling should be applied to all diagnostic patient samples. However where this is not possible the presence of a hyperdiploid clone, and particularly the presence of trisomy 1, should prompt further investigation by SNP profiling and/or *TP53* mutation testing. In addition, we have developed and validated a novel ploidy classifier to assist SNP array interpretation particularly in situations where the pattern is ambiguous. This novel approach is applicable to other cancers where large ploidy shifts define prognostically important subtypes, for example, multiple myeloma.^39^ As the majority of ALL treatment protocols assign patients with HoTr to high‐risk therapy the accurate detection of this subgroup should be considered standard‐of‐care for all patients with ALL.

## CONFLICT OF INTEREST

The authors declare no potential conflict of interest.

## AUTHOR CONTRIBUTIONS

Conception and Design: Thomas Creasey, Anthony V. Moorman. Collection and assembly of data: Thomas Creasey, Claire Schwab, Kathryn Watts, Gavin Cuthbert. Data analysis and interpretation: Thomas Creasey, Amir Enshaei, Anthony V. Moorman. Validation cohort and interpretation: Karin Nebral, Oskar A. Haas. Financial support: Anthony V. Moorman, Christine J. Harrison, John Moppett, Adele K. Fielding. Administrative support: Christine J. Harrison, Anthony V. Moorman. Provision of study materials or patients: Ajay Vora, John Moppett, Christine J. Harrison, Adele K. Fielding. Manuscript writing: Thomas Creasey, Anthony V. Moorman. Final approval of manuscript: All authors.

## Supporting information


**Supplementary Figure 1** Copy number status of individual chromosomes as reported in karyotype of high hyperdiploid cases with complete cytogenetic data (*n* = 13). Cases with discrepant cytogenetic and SNP array findings are not shown.
**Supplementary Figure 2**: Representative SNP array whole genome view of typical high hyperdiploid ALL case. (A) Log2 ratio trace (top) and B‐allele frequency trace (bottom). (B) Table detailing whole chromosomal log2 ratios for each chromosome 1–22 and whether B‐allele frequency indicates loss of heterozygosity (Y/N). The case demonstrates classic pattern of high hyperdiploidy where chromosomes with the lowest log2 ratio possess a normal disomic component of SNPs on B‐allele frequency (BB, AB, and AA alleles) and chromosomes with higher log2 ratio possess a trisomic pattern of SNPs (BBB, ABB, AAB, and AAA alleles) on B‐allele frequency.
**Supplementary Figure 3**: Copy number status of individual chromosomes as reported in karyotype of low hypodiploid and near triploid cases. Cases with low hypodiploid clones (*n* = 19) (lower panel), cases with near triploid clones (*n* = 23) (upper panel). Low hypodiploid clones included cases with only a low hypodiploid clone detected (*n* = 11) and cases with both low hypodiploid and near triploid clones (*n* = 8). Similarly near triploid clones included cases with near triploid clone only (*n* = 15) and cases with both low hypodiploid and near triploid clones (*n* = 7). Cases with discrepant cytogenetic and SNP array findings are not shown.
Cases with discrepant genetic subgroups by karyotype and SNP array
**Supplementary Figure 4**: Patient 26 910 cytogenetically classified as high hyperdiploidy and SNP array consistent with low hypodiploidy (blast percentage 68%). SNP array demonstrates loss of heterozygosity and reduced log2 ratio in chromosomes 3, 4, 7, 8, 9, 13, 15, 16, 17, 20. Chromosomes 1, 2, 6, 10, 11, 12, 18, 19 demonstrate an elevated log2 ratio with a B allele frequency consistent with trisomic pattern of SNPs. Chromosomes 21 and 22 show the highest log2 ratios and B‐allele frequency consistent with tetrasomy. The overall pattern is highly suggestive of duplicated low hypodiploid ALL. Using standardized log2 ratios, this case clustered with low hypodiploid/near triploid samples and the decision tree classifier assigned it to the HoTr node.
**Supplementary Figure 5**: Patient 29 491 cytogenetically classified as high hyperdiploidy and SNP array consistent with low hypodiploidy (blast percentage 90%). SNP array demonstrates Loss of heterozygosity and reduced log2 ratio in chromosomes 3, 4, 5, 7, 8, 9, 13, 15, 16, 17, 20. Chromosomes 1, 2, 6, 10, 11, 14, 18, 19, 22 demonstrate an elevated log2 ratio with a B allele frequency consistent with trisomic pattern of SNPs. Chromosomes 12 and 21 have the highest log2 ratios and B‐allele frequency consistent with tetrasomy. The overall pattern is highly suggestive of duplicated low hypodiploid ALL. Using standardized log2 ratios, this case clustered with low hypodiploid/near triploid samples and the decision tree classifier assigned it to the HoTr node.
**Supplementary Figure 6**: Patient 27 058 cytogenetically classified as HoTr and SNP array consistent with high hyperdiploidy (blast percentage unknown). SNP array demonstrates reduced log2 ratio in chromosomes 7, 9, 13, 15, 19, 20 but there is a preserved disomic and normal heterozygous complement of SNPs on B‐allele frequency with no loss of heterozygosity. Chromosomes 3, 4, 5, 6, 8, 10, 11, 12, 17, 18, 22 have increased log2 ratio and B‐allele frequency consistent with trisomies. Chromosome 21 has the highest log 2 ratio and B‐allele frequency consistent with pentasomy. Overall these findings rule out masked low hypodiploidy. Using standardized log2 ratios, this case clustered with high hyperdiploid samples and the decision tree classifier assigned it to a high hyperdiploid node.
**Supplementary Figure 7**: Patient 24 805 cytogenetically classified as high hyperdiploidy and SNP array showing largely normal profile (blast percentage 39%). This case had atypical chromosomal gains for HeH (including absence of +14) and therefore did not cluster with other high hyperdiploid samples. This case was put into a non‐ploidy node by the decision tree classifier.
**Supplementary Figure 8**: Patient 28 893 cytogenetically classified as near triploidy/masked low hypodiploidy subsequently shown to have *IGH‐CRLF2* (blast percentage 95%). Despite high reported blast percentage, appearances suggest contamination with non‐leukaemic DNA. Subtle abnormalities in BAF are visible in chromosomes 2, 3, 4, 6, 7, 8, 9, 11, 12, 13, 15. Case did not cluster with low hypodiploid/near triploid samples and further testing revealed *IGH‐CRLF2* fusion by FISH and *JAK2 p.T875N* mutation, confirming an alternative primary genetic abnormality.
**Supplementary Figure 9**: PCA of standardized of whole chromosomal log2 ratios labeled by SNP array platform. No SNP array platform batch effect was demonstrated between the standardized whole chromosome log2 ratios derived from Illumina CytoSNP 850k (*n* = 126) and Affymetrix CytoScan HD (*n* = 34) arrays.
**Supplementary Figure 10**: CART analysis performed of HoTr and HeH cases only including (A) and excluding (B) cases where SNP array appearances were inconclusive. Chromosome 1 remains the best predictor of HoTr versus HeH status irrespective of the inclusion of visually inconclusive arrays (n = 17), with very similar discriminating standardized log2 ratio values. Findings support the reliability of the standardization procedure in permitting CART analysis of visually inconclusive SNP arrays.
**Supplementary Figure 11**: Boxplot of log2 ratios by chromosome. Raw values (top panel) and standardized values (bottom panel). Cases labeled according to cytogenetic subgroup at diagnosis. Widest separation of low hypodiploid and high hyperdiploid cases is seen with standardized log2 ratio of chromosome 1.
**Supplementary Figure 12**: Receiver operating characteristic (ROC) curves of CART performance based on combined discovery and validation cohort. Each ploidy subgroup was assessed against the two others.

**Appendix S1**: Supporting Information

**Supplementary Table 1** List of gene covered by panel
**Supplementary Table 2**: List of cases with karyotypes, selected FISH results and classification categories.
**Supplementary Table 3**: Details of TP53 mutations identified
**Supplementary Table 4**: Whole chromosome log2 ratios for all cases in discovery cohort
**Supplementary Table 5**: Whole chromosome log2 ratios for all cases in validation cohort

## Data Availability

The data that support the findings of this study are available in the supplementary material of this article.
