# Supplementary material for: Single nucleotide polymorphism array‐based signature of low hypodiploidy in acute lymphoblastic leukemia
Source: Genes Chromosomes Cancer. 2021 May 17;60(9):604–15. doi: 10.1002/gcc.22956 (PMC8600946; doi:10.1002/gcc.22956)
Supplement: Supplementary file 1 — Supplementary Figure 1 Copy number status of individual chromosomes as reported in karyotype of high hyperdiploid cases with complete cytogenetic data (n = 13). Cases with discrepant cytogenetic and SNP array findings are not shown. Supplementary Figure 2: Representative SNP array whole genome view of typical high hyperdiploid ALL case. (A) Log2 ratio trace (top) and B‐allele frequency trace (bottom). (B) Table detailing whole chromosomal log2 ratios for each chromosome 1–22 and whether B‐allele frequency indicates loss of heterozygosity (Y/N). The case demonstrates classic pattern of high hyperdiploidy where chromosomes with the lowest log2 ratio possess a normal disomic component of SNPs on B‐allele frequency (BB, AB, and AA alleles) and chromosomes with higher log2 ratio possess a trisomic pattern of SNPs (BBB, ABB, AAB, and AAA alleles) on B‐allele frequency. Supplementary Figure 3: Copy number status of individual chromosomes as reported in karyotype of low hypodiploid and near triploid cases. Cases with low hypodiploid clones (n = 19) (lower panel), cases with near triploid clones (n = 23) (upper panel). Low hypodiploid clones included cases with only a low hypodiploid clone detected (n = 11) and cases with both low hypodiploid and near triploid clones (n = 8). Similarly near triploid clones included cases with near triploid clone only (n = 15) and cases with both low hypodiploid and near triploid clones (n = 7). Cases with discrepant cytogenetic and SNP array findings are not shown. Cases with discrepant genetic subgroups by karyotype and SNP array Supplementary Figure 4: Patient 26 910 cytogenetically classified as high hyperdiploidy and SNP array consistent with low hypodiploidy (blast percentage 68%). SNP array demonstrates loss of heterozygosity and reduced log2 ratio in chromosomes 3, 4, 7, 8, 9, 13, 15, 16, 17, 20. Chromosomes 1, 2, 6, 10, 11, 12, 18, 19 demonstrate an elevated log2 ratio with a B allele frequency consistent with trisomic pattern of SNP [file GCC-60-604-s001.docx]

**Chromosomal copy number states and typical representative SNP arrays**


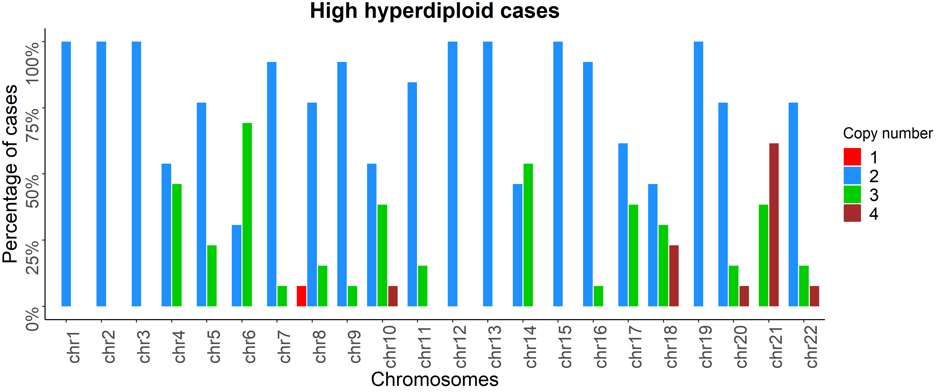


**Supplementary figure 1: Copy number status of individual chromosomes as reported in karyotype of high hyperdiploid cases with complete cytogenetic data (n=13)**. Cases with discrepant cytogenetic and SNP array findings are not shown.


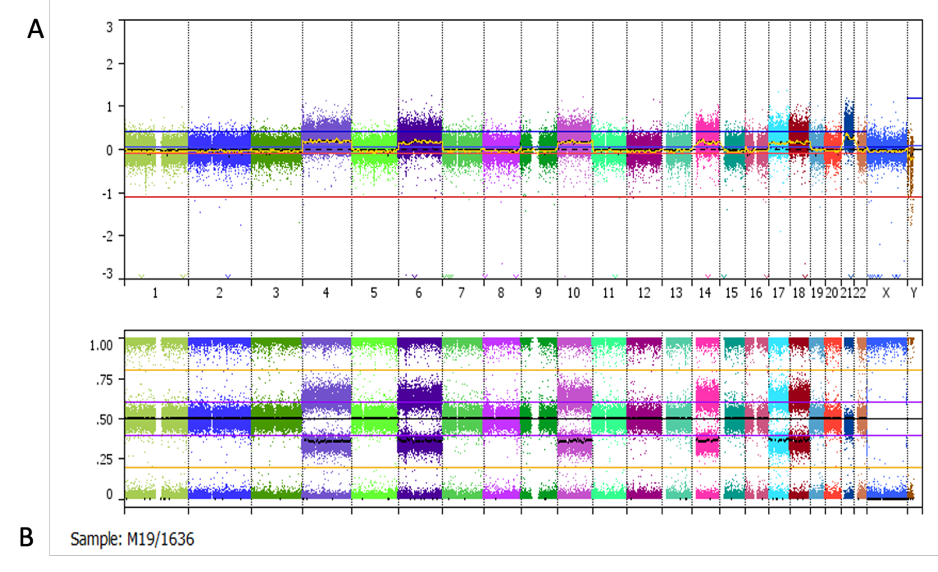


**
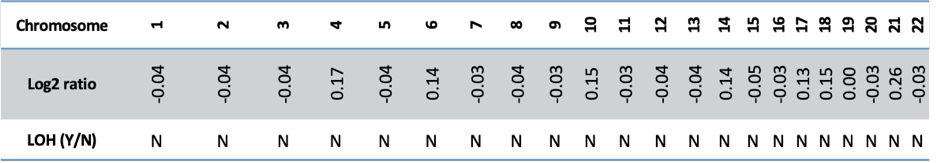
Supplementary figure 2: Representative SNP array whole genome view of typical high hyperdiploid ALL case**. (A) Log2 ratio trace (top) and B-allele frequency trace (bottom). (B) Table detailing whole chromosomal log2 ratios for each chromosome 1-22 and whether B-allele frequency indicates loss of heterozygosity (Y/N). The case demonstrates classic pattern of high hyperdiploidy where chromosomes with the lowest log2 ratio possess a normal disomic component of SNPs on B-allele frequency (BB, AB and AA alleles) and chromosomes with higher log2 ratio possess a trisomic pattern of SNPs (BBB, ABB, AAB and AAA alleles) on B-allele frequency.


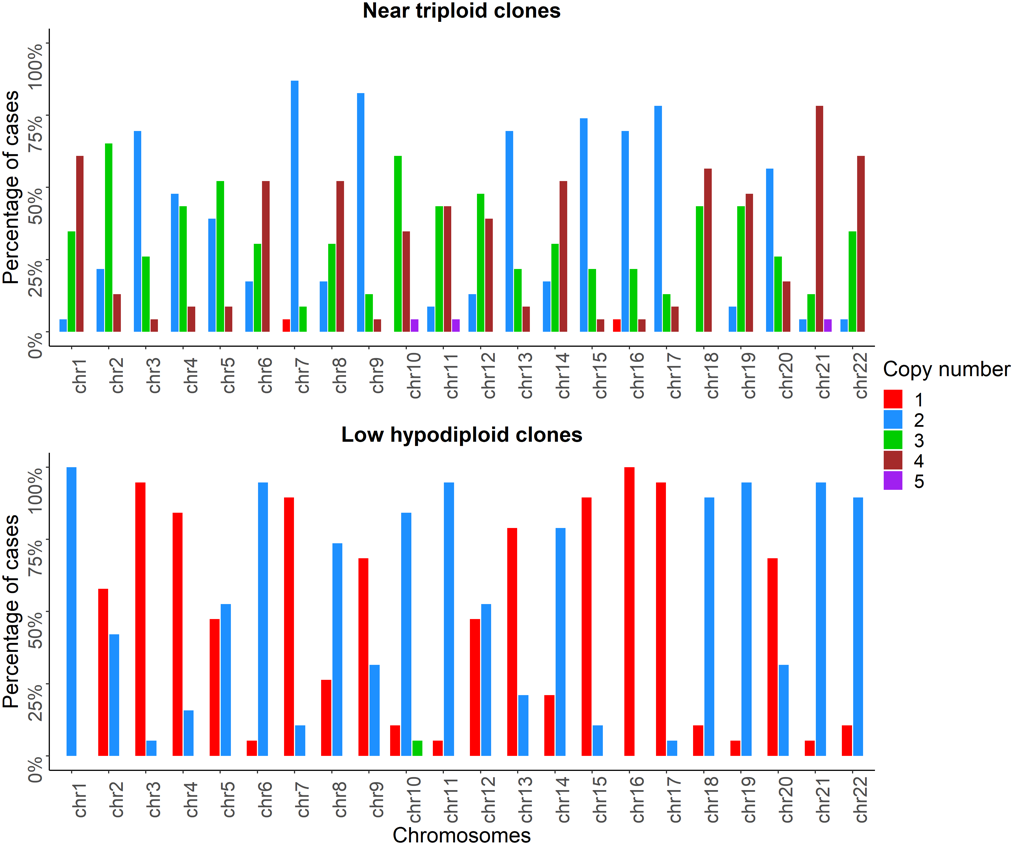


**Supplementary figure 3: Copy number status of individual chromosomes as reported in karyotype of low hypodiploid and near triploid cases.** Cases with low hypodiploid clones (n=19) (lower panel), cases with near triploid clones (n=23) (upper panel). Low hypodiploid clones included cases with only a low hypodiploid clone detected (n=11) and cases with both low hypodiploid and near triploid clones (n=8). Similarly near triploid clones included cases with near triploid clone only (n=15) and cases with both low hypodiploid and near triploid clones (n=7). Cases with discrepant cytogenetic and SNP array findings are not shown.

**Cases with discrepant genetic subgroups by karyotype and SNP array**

**
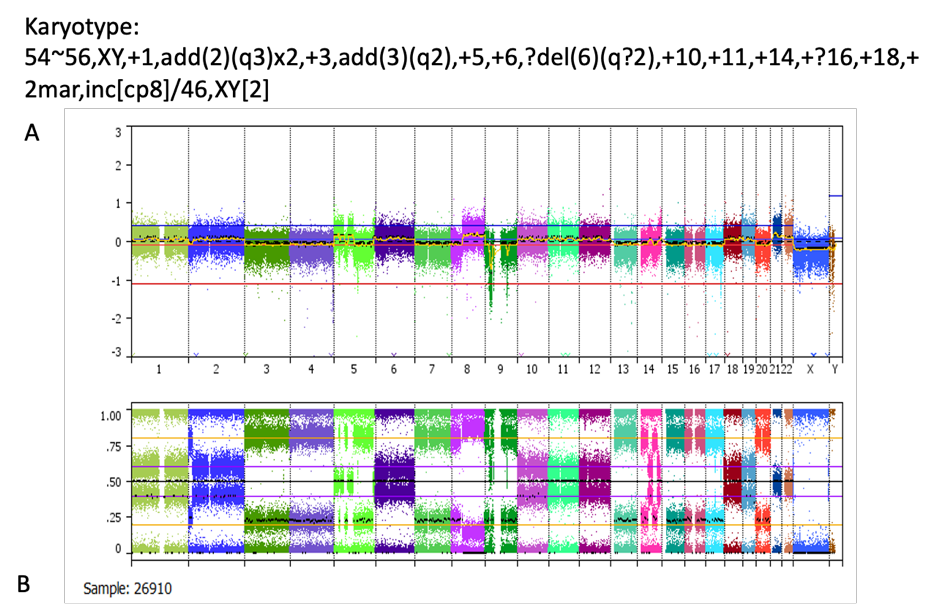

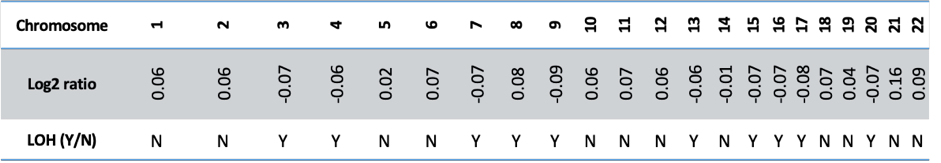
**

**Supplementary figure 4: Patient 26910 cytogenetically classified as high hyperdiploidy and SNP array consistent with low hypodiploidy (blast percentage 68%).** SNP array demonstrates loss of heterozygosity and reduced log2 ratio in chromosomes 3, 4, 7, 8, 9, 13, 15, 16, 17, 20. Chromosomes 1, 2, 6, 10, 11, 12, 18, 19 demonstrate an elevated log2 ratio with a B allele frequency consistent with trisomic pattern of SNPs. Chromosomes 21 and 22 show the highest log2 ratios and B-allele frequency consistent with tetrasomy. The overall pattern is highly suggestive of duplicated low hypodiploid ALL. Using standardised log2 ratios, this case clustered with low hypodiploid/near triploid samples and the decision tree classifier assigned it to the HoTr node.


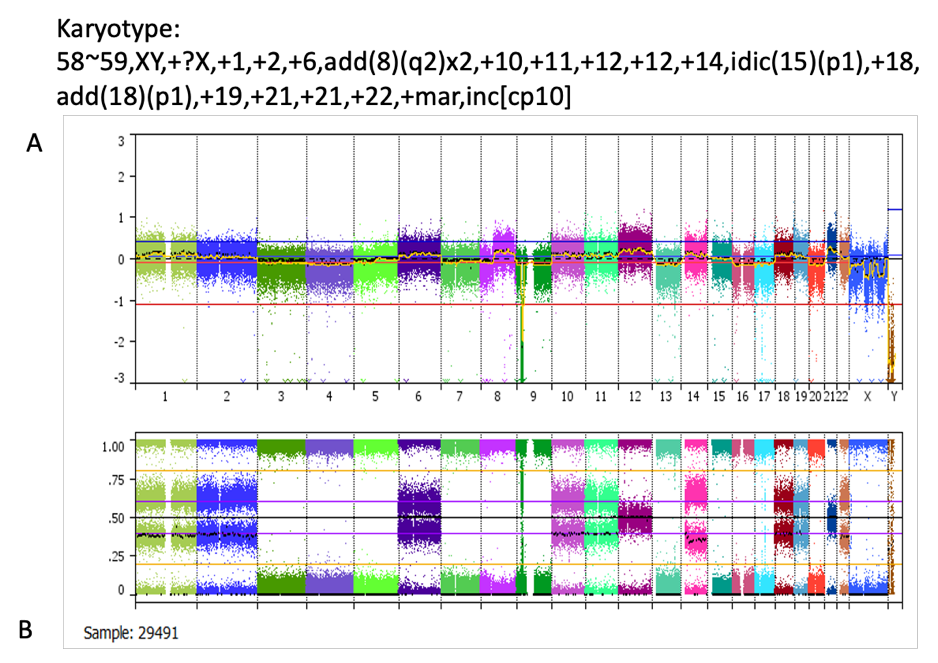

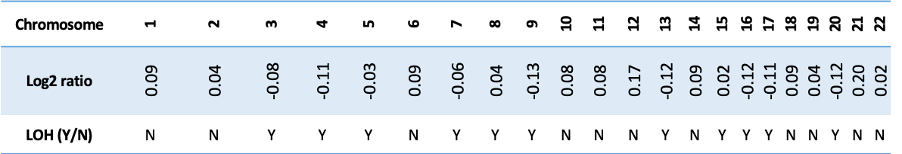


**Supplementary figure 5: Patient 29491 cytogenetically classified as high hyperdiploidy and SNP array consistent with low hypodiploidy (blast percentage 90%).** SNP array demonstrates Loss of heterozygosity and reduced log2 ratio in chromosomes 3, 4, 5, 7, 8, 9, 13, 15, 16, 17, 20. Chromosomes 1, 2, 6, 10, 11, 14, 18, 19, 22 demonstrate an elevated log2 ratio with a B allele frequency consistent with trisomic pattern of SNPs. Chromosomes 12 and 21 have the highest log2 ratios and B-allele frequency consistent with tetrasomy. The overall pattern is highly suggestive of duplicated low hypodiploid ALL. Using standardised log2 ratios, this case clustered with low hypodiploid/near triploid samples and the decision tree classifier assigned it to the HoTr node.


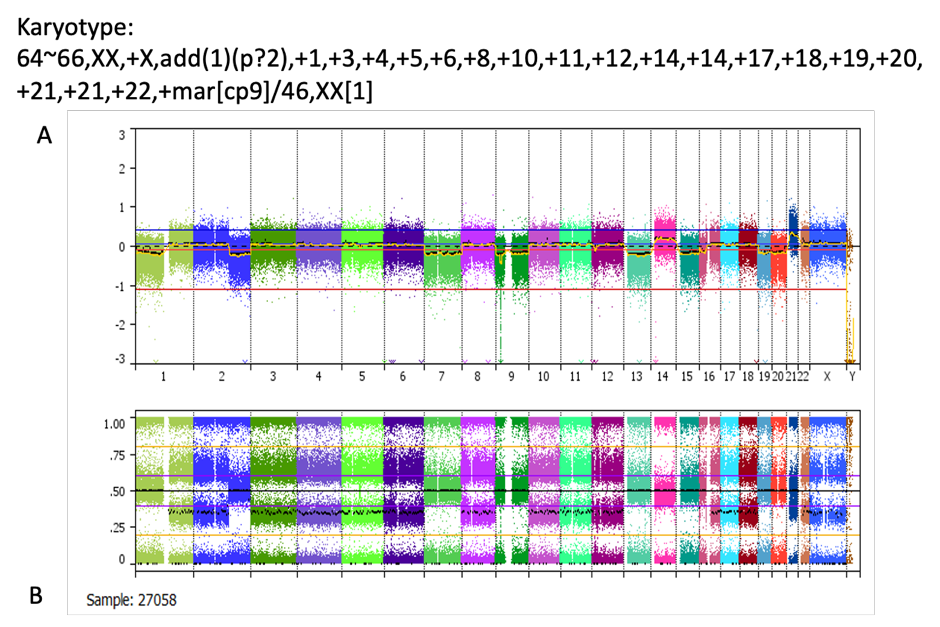

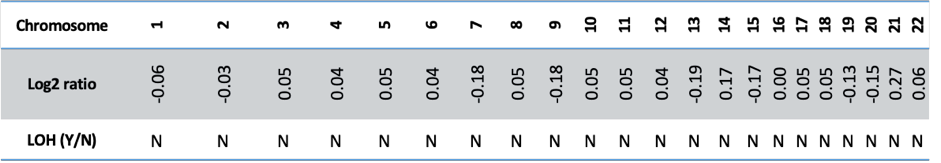


**Supplementary figure 6: Patient 27058 cytogenetically classified as HoTr and SNP array consistent with high hyperdiploidy (blast percentage unknown).** SNP array demonstrates reduced log2 ratio in chromosomes 7, 9, 13, 15, 19, 20 but there is a preserved disomic and normal heterozygous complement of SNPs on B-allele frequency with no loss of heterozygosity. Chromosomes 3, 4, 5, 6, 8, 10, 11, 12, 17, 18, 22 have increased log2 ratio and B-allele frequency consistent with trisomies. Chromosome 21 has the highest log 2 ratio and B-allele frequency consistent with pentasomy. Overall these findings rule out masked low hypodiploidy. Using standardised log2 ratios, this case clustered with high hyperdiploid samples and the decision tree classifier assigned it to a high hyperdiploid node.


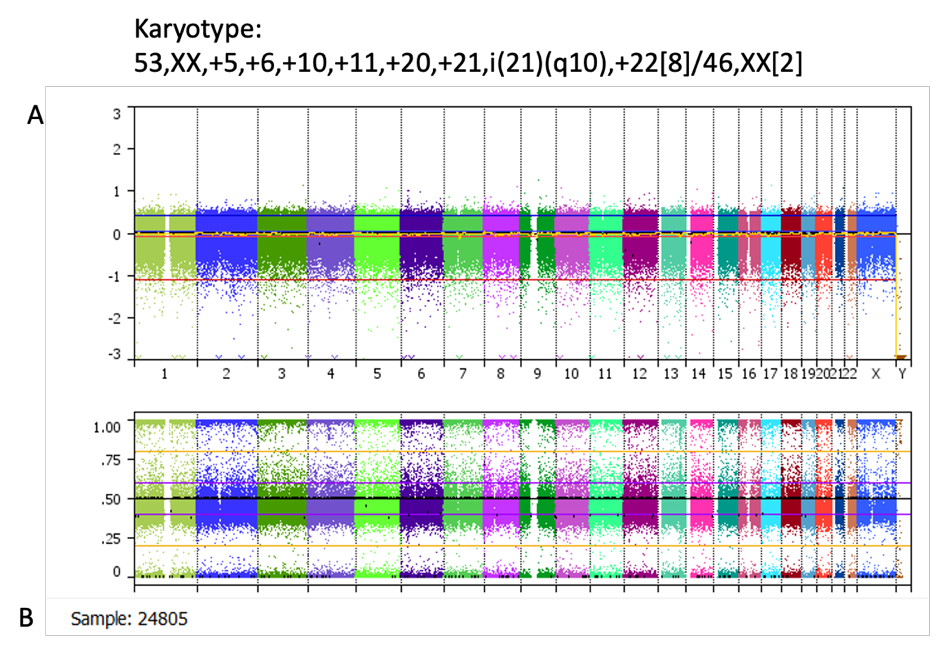

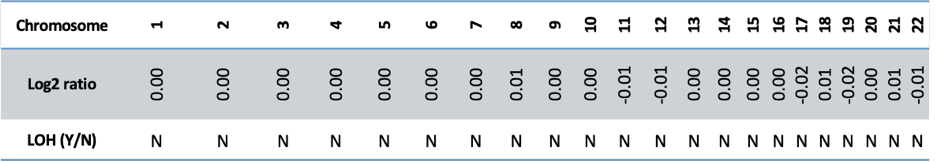


**Supplementary figure 7: Patient 24805 cytogenetically classified as high hyperdiploidy and SNP array showing largely normal profile (blast percentage 39%).** This case had atypical chromosomal gains for HeH (including absence of +14) and therefore did not cluster with other high hyperdiploid samples. This case was put into a non-ploidy node by the decision tree classifier.


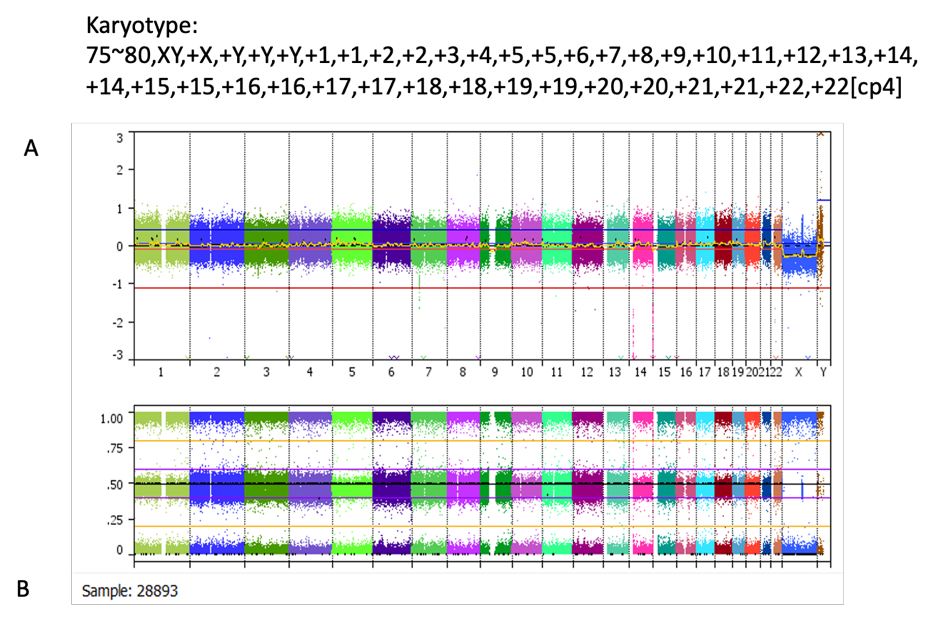


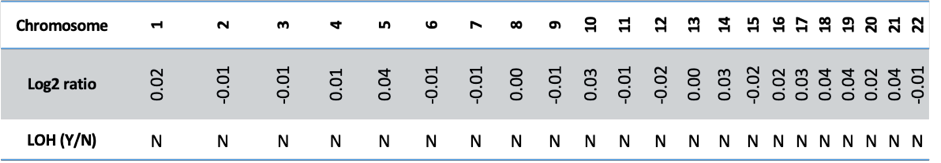


**Supplementary figure 8: Patient 28893 cytogenetically classified as near triploidy/masked low hypodiploidy subsequently shown to have *IGH-CRLF2* (blast percentage 95%).** Despite high reported blast percentage, appearances suggest contamination with non-leukaemic DNA. Subtle abnormalities in BAF are visible in chromosomes 2, 3, 4, 6, 7, 8, 9, 11, 12, 13, 15. Case did not cluster with low hypodiploid/near triploid samples and further testing revealed *IGH-CRLF2* fusion by FISH and *JAK2 p.T875N* mutation, confirming an alternative primary genetic abnormality.


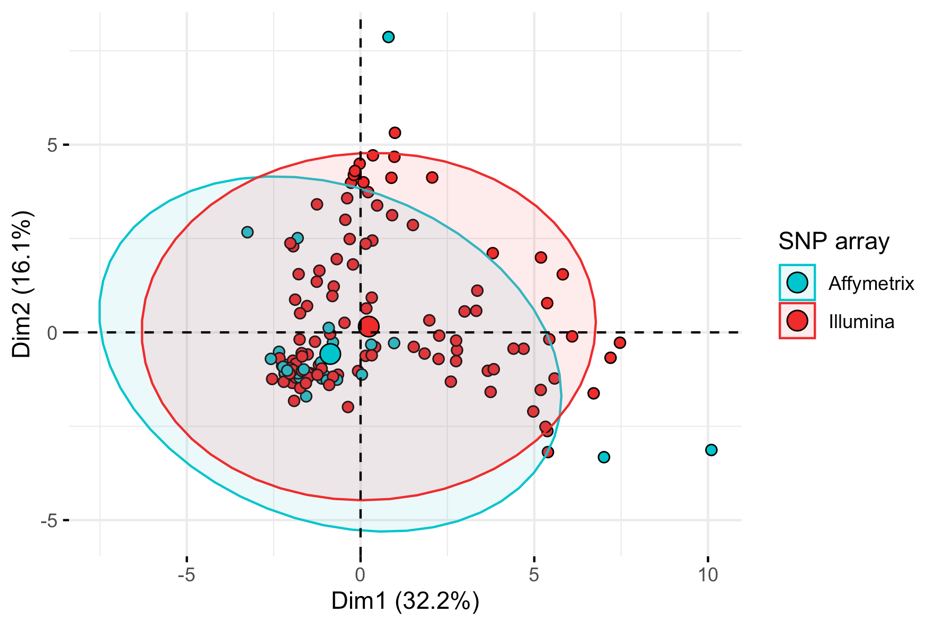


**Supplementary figure 9: PCA of standardized of whole chromosomal log2 ratios labelled by SNP array platform.** No SNP array platform batch effect was demonstrated between the standardized whole chromosome log2 ratios derived from Illumina CytoSNP 850k (n=126) and Affymetrix CytoScan HD (n=34) arrays.


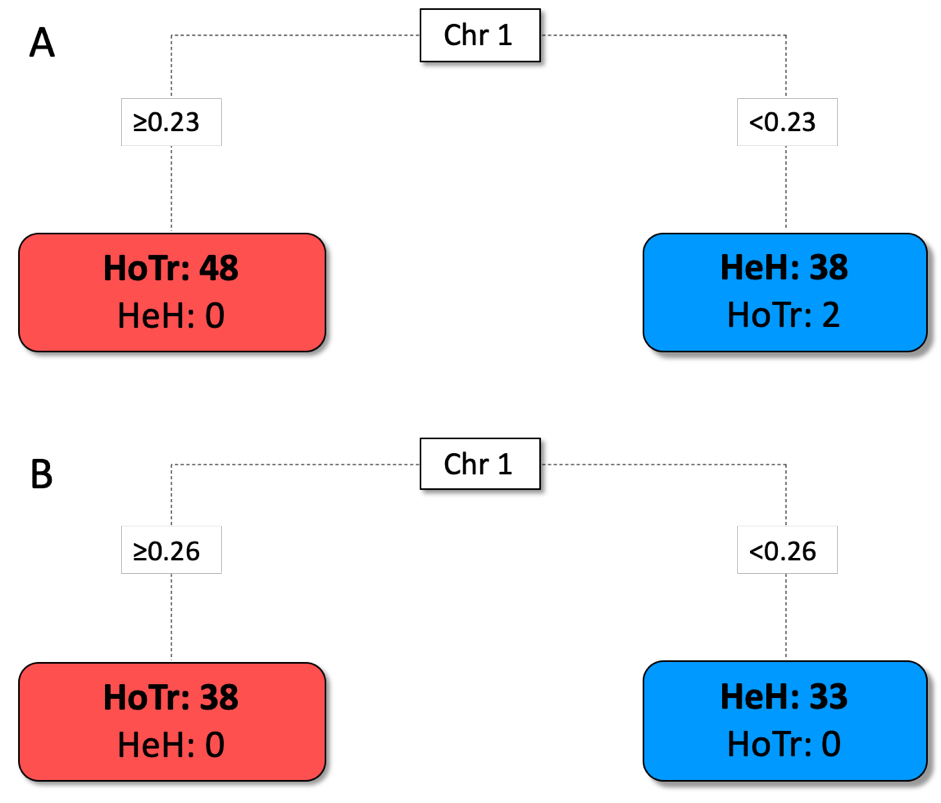


**Supplementary figure 10: CART analysis performed of HoTr and HeH cases only including (A) and excluding (B) cases where SNP array appearances were inconclusive.** Chromosome 1 remains the best predictor of HoTr vs HeH status irrespective of the inclusion of visually inconclusive arrays (n=17), with very similar discriminating standardised log2 ratio values. Findings support the reliability of the standardisation procedure in permitting CART analysis of visually inconclusive SNP arrays.

**
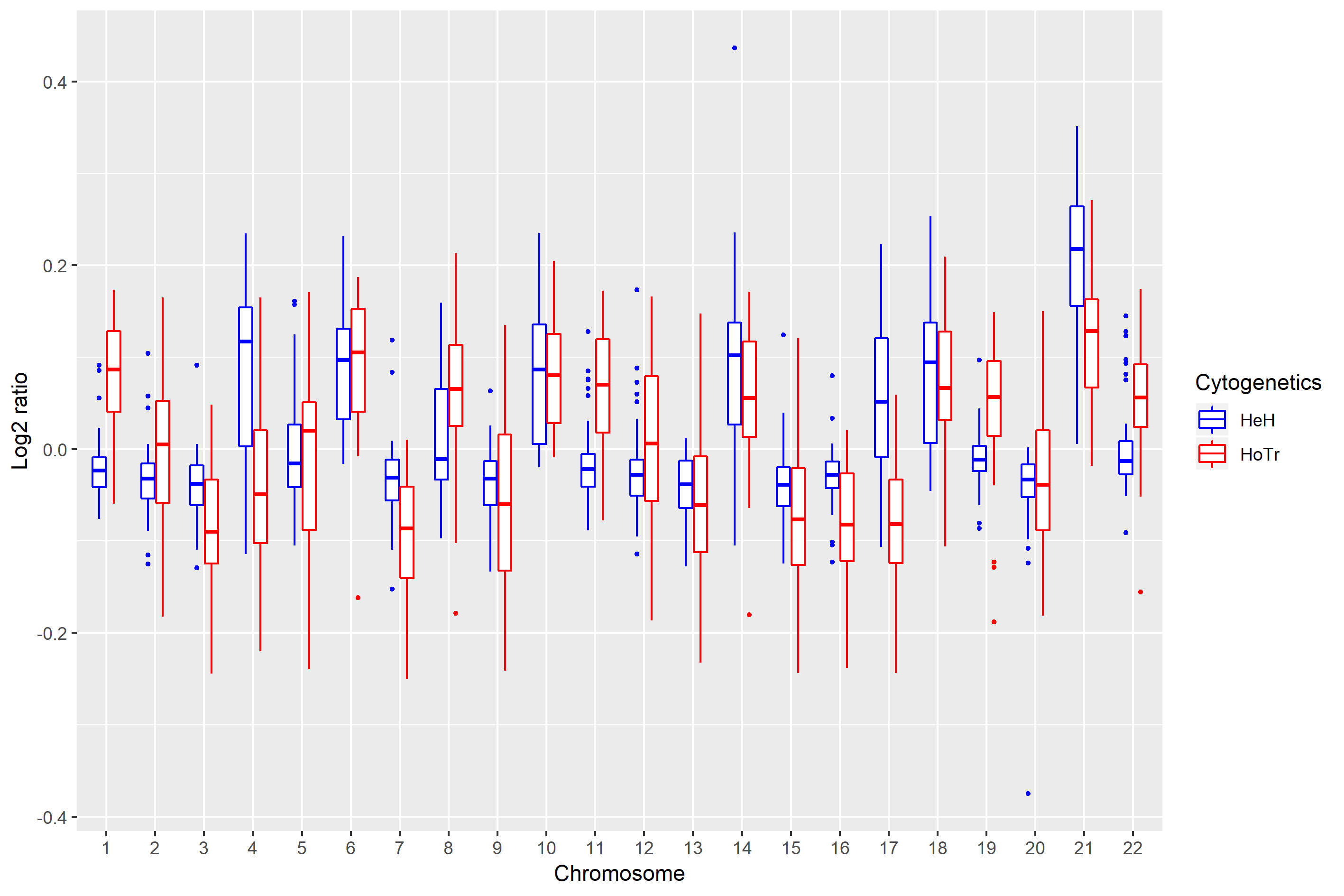

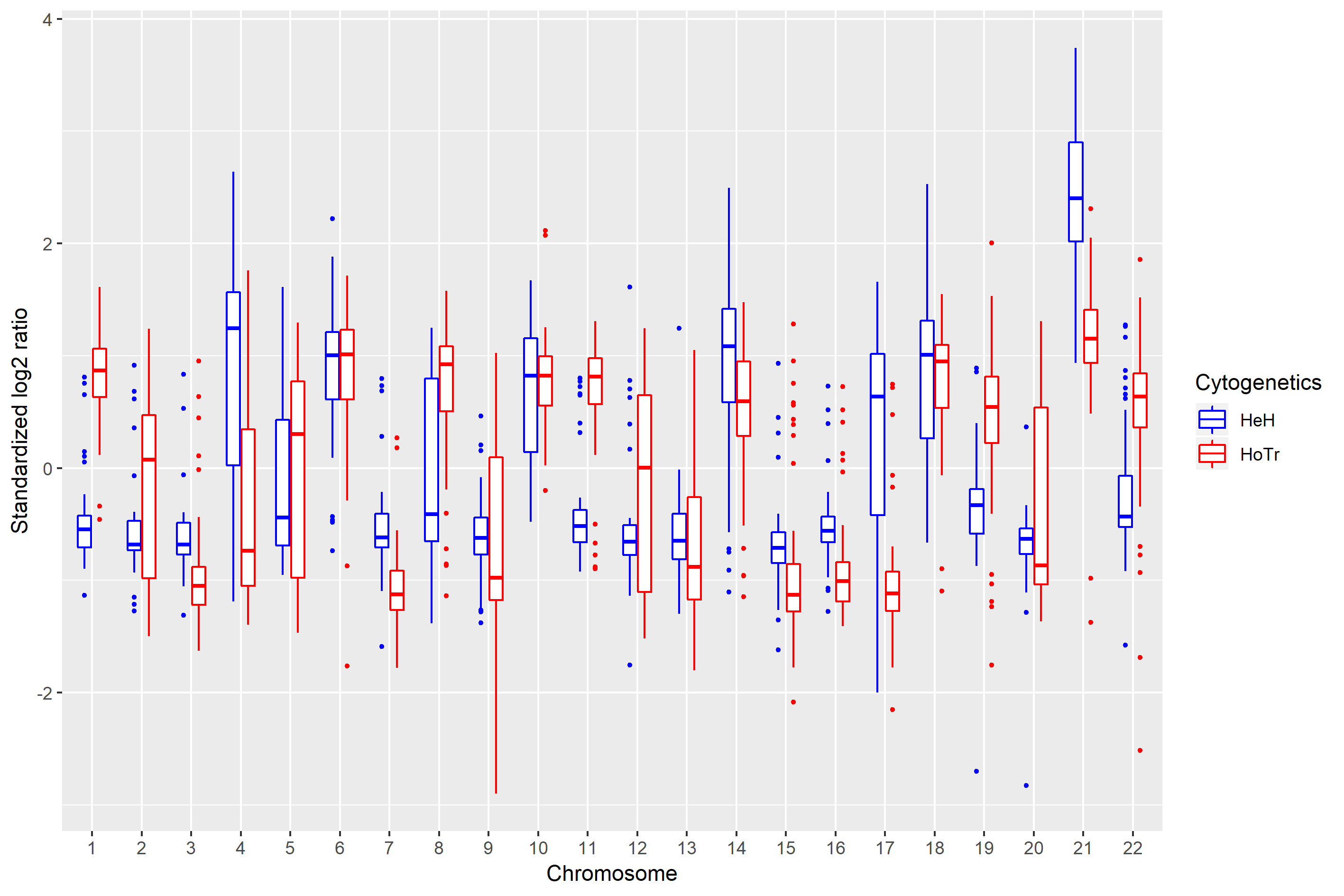
**

**Supplementary figure 11: Boxplot of log2 ratios by chromosome**. Raw values (top panel) and standardized values (bottom panel). Cases labelled according to cytogenetic subgroup at diagnosis. Widest separation of low hypodiploid and high hyperdiploid cases is seen with standardised log2 ratio of chromosome 1.


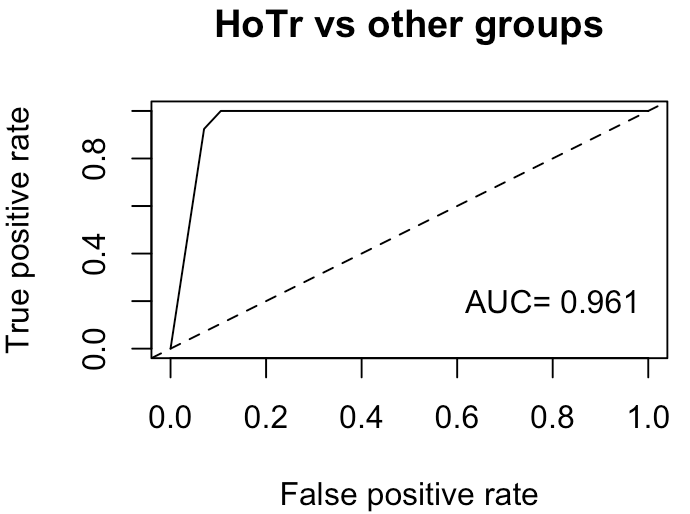


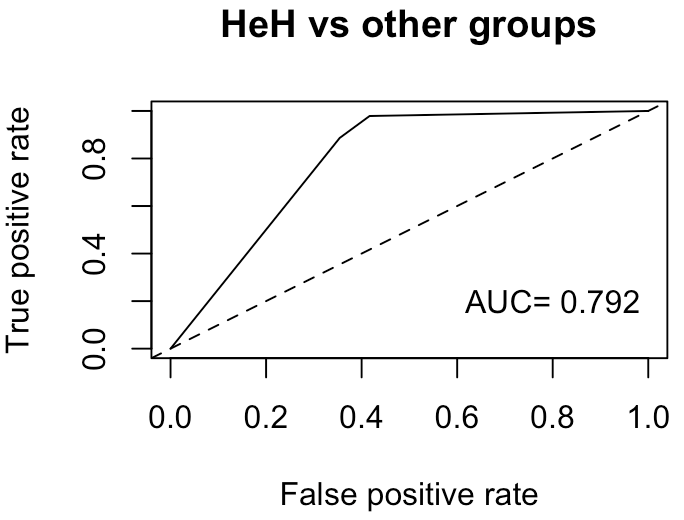


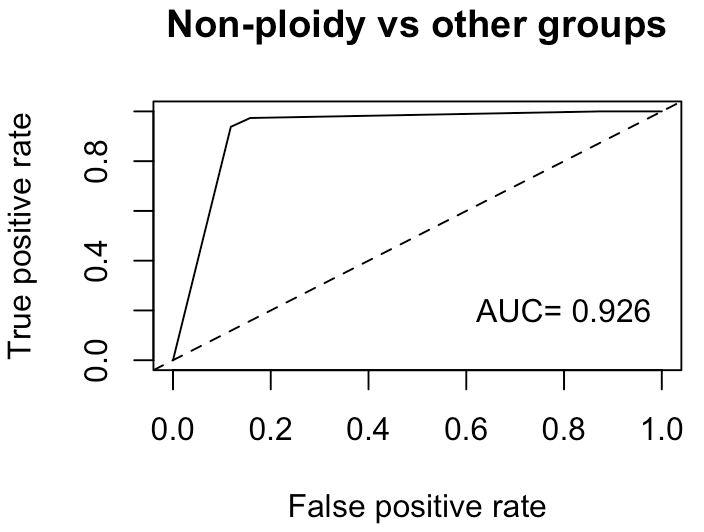


**Supplementary figure 12: Receiver operating characteristic (ROC) curves of CART performance based on combined discovery and validation cohort.** Each ploidy subgroup was assessed against the two others.
