# Supplementary material for: Single nucleotide polymorphism array‐based signature of low hypodiploidy in acute lymphoblastic leukemia
Source: Genes Chromosomes Cancer. 2021 May 17;60(9):604–15. doi: 10.1002/gcc.22956 (PMC8600946; doi:10.1002/gcc.22956)
Supplement: Supplementary file 2 — Appendix S1: Supporting Information [file GCC-60-604-s003.docx]

**Supplementary methods**

**Preparation of SNP array data**

Raw array data from Affymetrix (Santa Clara, CA, USA) arrays were loaded directly to Nexus Copy Number 10 (Biodiscovery, El Segundo, CA, USA) in the form of CEL files. Illumina (San Diego, CA, USA)-generated IDAT files first needed to be converted into a text-based format before being loaded to Nexus. To achieve this, the Illumina-specific SNP array software package – GenomeStudio 2.0 – was used, in accordance with the Nexus protocol for the analysis of Illumina arrays. The IDAT files were loaded to GenomeStudio 2.0 and then converted into text-based format by creating a Final_Report file. This was then loaded onto Nexus to visualise the data and perform copy number segmentation.

Systematic correction of the arrays was then performed. This is a recommended step in the analysis of SNP array data due to the waviness in the probe signals that can often be seen across the genome^1^. This is partly related to GC content as probes with high GC content will bind better to their target sequence, producing a higher signal intensity. As such, systematic correction was performed in Nexus using the recommended Illumina and Affymetrix correction files.

Nexus employs a hybrid segmentation algorithm termed Fast Adaptive States Segmentation Technique (FASST2). This is based on HMM-segmentation but does not assume fixed integer levels of copy number, and instead accepts a large number of potential copy number states falling between fixed integer levels.

**Creation of whole chromosomal log2 ratios**

Once SNP arrays were loaded to Nexus, manual whole chromosome segments were created using the “Add Regions” function. A new segment was created for each chromosome 1-22 in each sample. This removed all prior automated calls within each chromosome, replacing them with a single call spanning the entire length of the chromosome. Within the sample report table, the median log2 ratio of each segment is computed (‘probe median’), thereby generating 22 individual whole chromosomal log2 ratios for the sample. These values were transferred to a CSV file (supplementary table 4) for subsequent analysis in R version 4.0.3^2^.

**Whole chromosome log2 ratio standardization**

The scale of log2 ratio values for the copy number segments within a SNP array sample are influenced by a number of factors, including sample purity, clonal heterogeneity and SNP array platform. To account for these variables and therefore permit comparison of whole chromosome log2 ratios between samples, the whole chromosome log2 ratios were standardized within each sample using R-package BBmisc. This was performed using the normalize function (method = standardize), which transformed the whole chromosome log2 ratios within each sample, such that the median whole chromosome log2 ratio was 0 and the standard deviation across all 22 whole chromosome log2 ratio values was 1. These standardized whole chromosome log2 ratios were then used for all subsequent clustering and classification analyses.

**Next generation sequencing (NGS) analyses**

NGS was performed on selected samples where the cytogenetic subgroup conflicted with the SNP array findings. A SureSelect XT2 capture library (Agilent, Santa Clara, CA, USA ) was designed using the SureDesign web portal (<https://earray.chem.agilent.com/suredesign/>) to capture either exons or the full sequence of genes implicated in leukaemogenesis (supplementary table 1). DNA samples were first amplified using a REPLI-g mini kit (Qiagen, Hilden, Germany) to yield at least 1 ug of whole genome amplified DNA. Following amplification, double stranded DNA concentrations were measured using the Quant-iT Picogreen broad-range assay (Invitrogen, Carlsbad, CA, USA) and FLUOstar Omega microplate reader (BGM Labtech, Ortenberg, Germany). SureSelect XT2 libraries were then prepared using 1 ug of input DNA. Mechanical DNA shearing was performed on the Bioruptor pico sonication system (Diagenode, Liège, Belgium) inputting shearing settings as follow – time on: 7 seconds; time off: 90 seconds; number of cycles: 4 – to yield 800-1000 bp fragments. Sample quality and fragment size were then assessed on the 2100 Bioanalyzer (Agilent) to ensure that a fragment peak was visible around 800-1000 bp. Library prep was then performed according to the manufacturer’s protocol with two exceptions: i) the ratio of AMPure XP beads (Beckman Coulter, Brea, CA, USA): DNA was reduced to 0.7 to optimise bead-binding to the longer DNA fragments in the library and ii) The PCR amplification was performed using the Longamp Taq polymerase enzyme (New England Biolabs, Ipswich MA, USA), which is optimized for amplification of longer DNA fragments.

Each pooled library was sequenced using a mid-output kit on the Illumina NextSeq 550 with 100bp paired end reads. BAM files were generated and then deduplicated and re-aligned to the reference genome (hg19/GRCh37). As no germline DNA was available, variant calling was performed using the GATK HaplotypeCaller^3^. Ensembl VEP files were produced and calls with a population allele frequency ≥0.01 in the Exome Aggregation Consortium (ExAC) database^4^ were excluded as likely germline variants. All non-coding variants, synonymous variants, and those reported as both tolerated and benign in the SIFT^5^ and Polyphen^6^ databases respectively were also excluded. Calls with COSMIC identifiers were examined in the COSMIC database^7^ to identify known somatic mutations in cancer, specifically those in *TP53*.

**Decision tree classifier**

Classification and regression tree (CART) analysis is a supervised machine learning technique, whereby a dataset consisting of different classes is partitioned according to a set of variables. The variables that produce the clearest separation of the different classes are chosen by the model in a sequential manner to eventually obtain nodes that give the best separation of the input classes. This method can therefore be used effectively to produce an algorithm (decision tree) to classify a new case based on the same variables. Of the supervised machine-learning techniques used for classification, we selected CART due to its simplicity and high interpretability. Our priority was to produce a classifier that could be evaluated and used in a clinical diagnostic setting.

Accordingly, standardized whole chromosome log2 ratios were used to aid the accurate classification of ploidy status (HoTr, HeH or non-ploidy) based on a CART analysis with the aim of creating a decision tree classifier. Initially, all HoTr and HeH cases in the cohort were re-classified based on the most probable genetic subgroup using all available data from cytogenetics, SNP arrays and *TP53* status. Where the SNP profile showed the clear LOH-LCN pattern, the case was categorised as HoTr, even if this conflicted with the initial cytogenetic subgroup. Similarly, if the SNP array demonstrated the HET-CNG pattern consistent with chromosomal gains, the case was classified as HeH. Whole chromosomal log2 ratios were created and standardised as detailed above.

Following any re-classification of HoTr or HeH discrepancies, the CART analysis was performed using R-package rpart (see supplementary table 4 for input dataset), and a decision tree was created based on the standardised whole chromosomal log2 ratios. The rpart function was used with the ploidy subgroup as the response variable and all whole chromosome log2 ratios as the predictor variables.

The performance of the CART model was then assessed using internal 10-fold cross-validation in R-package caret. This involved internal random-partitioning of the dataset into 10 subsets. Each subset was held out in turn and the remainder of the dataset was used to train the decision tree. The accuracy of the decision tree in classifying cases in the held out subset was then assessed. As each subset was held out in turn, this generated 10 independent validations and the mean accuracy of the model was reported.

1. Diskin SJ, Li M, Hou C, et al. Adjustment of genomic waves in signal intensities from whole-genome SNP genotyping platforms. *Nucleic acids research*. 2008;36(19):e126-e126.

2. R Core Team. R: A language and environment for statistical computing. Vienna, Austria: R Foundation for Statistical Computing; 2020.

3. Poplin R, Ruano-Rubio V, DePristo MA, et al. Scaling accurate genetic variant discovery to tens of thousands of samples. *bioRxiv*. 2017:201178.

4. Karczewski KJ, Weisburd B, Thomas B, et al. The ExAC browser: displaying reference data information from over 60 000 exomes. *Nucleic acids research*. 2017;45(D1):D840-D845.

5. Sim N-L, Kumar P, Hu J, Henikoff S, Schneider G, Ng PC. SIFT web server: predicting effects of amino acid substitutions on proteins. *Nucleic Acids Research*. 2012;40(W1):W452-W457.

6. Adzhubei IA, Schmidt S, Peshkin L, et al. A method and server for predicting damaging missense mutations. *Nature methods*. 2010;7(4):248-249.

7. Tate JG, Bamford S, Jubb HC, et al. COSMIC: the Catalogue Of Somatic Mutations In Cancer. *Nucleic acids research*. 2019;47(D1):D941-D947.
